# Extracorporeal cardiopulmonary resuscitation for cardiogenic shock in a Jehovah’s witness

**DOI:** 10.1007/s10047-026-01591-6

**Published:** 2026-09-28

**Authors:** Derek K. Afflu, Louis F. Chai, Hiromu Kehara, Sean M. Baskin, Yoshiya Toyoda, Roh Yanagida

**Affiliations:** Lewis Katz School of Medicine at Temple University, Philadelphia, PA USA

**Keywords:** Extracorporeal membrane oxygenation, Bloodless medicine, Mechanical circulatory support, Cardiopulmonary resuscitation, Jehovah’s witness

## Abstract

Veno-arterial (V-A) and veno-venous (V-V) extracorporeal membrane oxygenation (ECMO) are frequently employed in the management of cardiopulmonary failure in critically ill patients. Utilization, however, is controversial in the Jehovah’s Witness (JW) population as temporary mechanical circulatory support (tMCS) typically is associated with blood loss requiring transfusions. Despite this fact, use of ECMO is still feasible in this population while avoiding blood transfusions and there have been few reported cases in the literature. However, its use as part of cardiopulmonary resuscitation is limited. Multiple strategies have been employed to limit blood loss and maximize endogenous hematopoiesis to assist in maintaining normal physiology while utilizing tMCS. Though these tactics are most useful when applied in the preintervention setting, they are applicable in the emergency setting including extracorporeal cardiopulmonary resuscitation (ECPR). This can be achieved with conscientious cannulation technique and circuit management. Presented here is a case report of the successful use of V-A ECMO in the resuscitation for a JW, avoiding blood product transfusions. The protocol utilized to eliminate the need for blood transfusions is also discussed. This approach adds to the growing body of evidence emphasizing that the use of tMCS as part of bloodless medicine practice is feasible, even in the emergency setting.

## Introduction

Extracorporeal membrane oxygenation (ECMO) is a common form of temporary mechanical circulatory support (tMCS) for patients with failing cardiopulmonary function. Management of patients on ECMO is commonly associated with blood product transfusions due to hemolysis or coagulopathy-associated bleeding [1].

Jehovah’s Witnesses (JWs) refuse transfusions, based on religious belief, creating a unique challenge in providing care, particularly in life threatening scenarios that may require ECMO [1, 2]. However, being a JW is not an absolute contraindication to the use of ECMO. We present a case of a JW who underwent extracorporeal cardiopulmonary resuscitation (ECPR) and was successfully weaned off ECMO and discharged without blood transfusions.

## Case report

All procedures in this study were conducted according to the principles of the Declaration of Helsinki. No Institutional Review Board/Ethics Board approval was required given the case report nature of the manuscript. Informed consent from this patient in writing before all procedures were performed.

A 61-year-old male JW with a history of transcatheter aortic valve replacement (TAVR) for severe aortic stenosis (AS) presented with worsening shortness of breath, orthopnea, and chest pain, consistent with progressive congestive heart failure. Acute coronary syndrome was ruled out based on absence of ST changes on EKG and a troponin leak. His co-morbidities included coronary artery disease requiring drug eluting stent (DES) to the left anterior descending artery (LAD) and end-stage renal disease requiring hemodialysis. Transthoracic echocardiogram (TTE) obtained showed severe bioprosthetic AS with mean gradient (MG) 43 mmHg and a peak velocity (V_max_) of 3.98 m/s. Left ventricular ejection fraction (LVEF) was 5–10%, from 35 to 40% one year prior. The reduction was suspected to be related to coronary stent occlusion. Cardiac catheterization revealed distal in-stent restenosis (ISR) of the LAD stent which was treated with angioplasty and a second DES coupled with an aspirin and clopidogrel load. Despite this intervention, the patient continued to have increased inotrope requirements over 24 h and was planned for urgent TAVR-in-TAVR to address his persistent cardiogenic shock. Prior to this, the patient developed pulseless electrical activity with maximum pharmacologic hemodynamic support. ACLS was initiated with rapid mobilization of the ECMO team for ECPR. During no-flow period of 1 min followed by a low-flow period of 44 min with total CPR time of 45 min, a 25 Fr drainage cannula in the right common femoral vein and a 17 Fr cannula in the left common femoral artery were percutaneously inserted. Manual pressure was always applied when exchanging dilators to minimize blood loss. A 6 French distal perfusion cannula was placed with a micropuncture needle.

Following cannulation, acceptable blood products for transfusion were confirmed with family. Cardiopulmonary bypass, ECMO, and cell saver use for procedures were also confirmed to be acceptable. He was started on 20,000 units of IV epoetin alpha-epbx daily, with additional 40,000 units on the day of V-A ECMO cannulation and TAVR, 1000mcg of enteric cobalamin daily, 2 mg of IV folic acid daily, and 125 mg of IV ferric gluconate daily per Hematology. Blood draws were minimized and clustered and done with pediatric collection tubes. Malnutrition was avoided to prevent anemia and coagulopathy. Given the severity of the patient’s valvular disease and recent DES placement, he was maintained on therapeutic heparin and dual antiplatelet therapy.

**Fig. 1:**
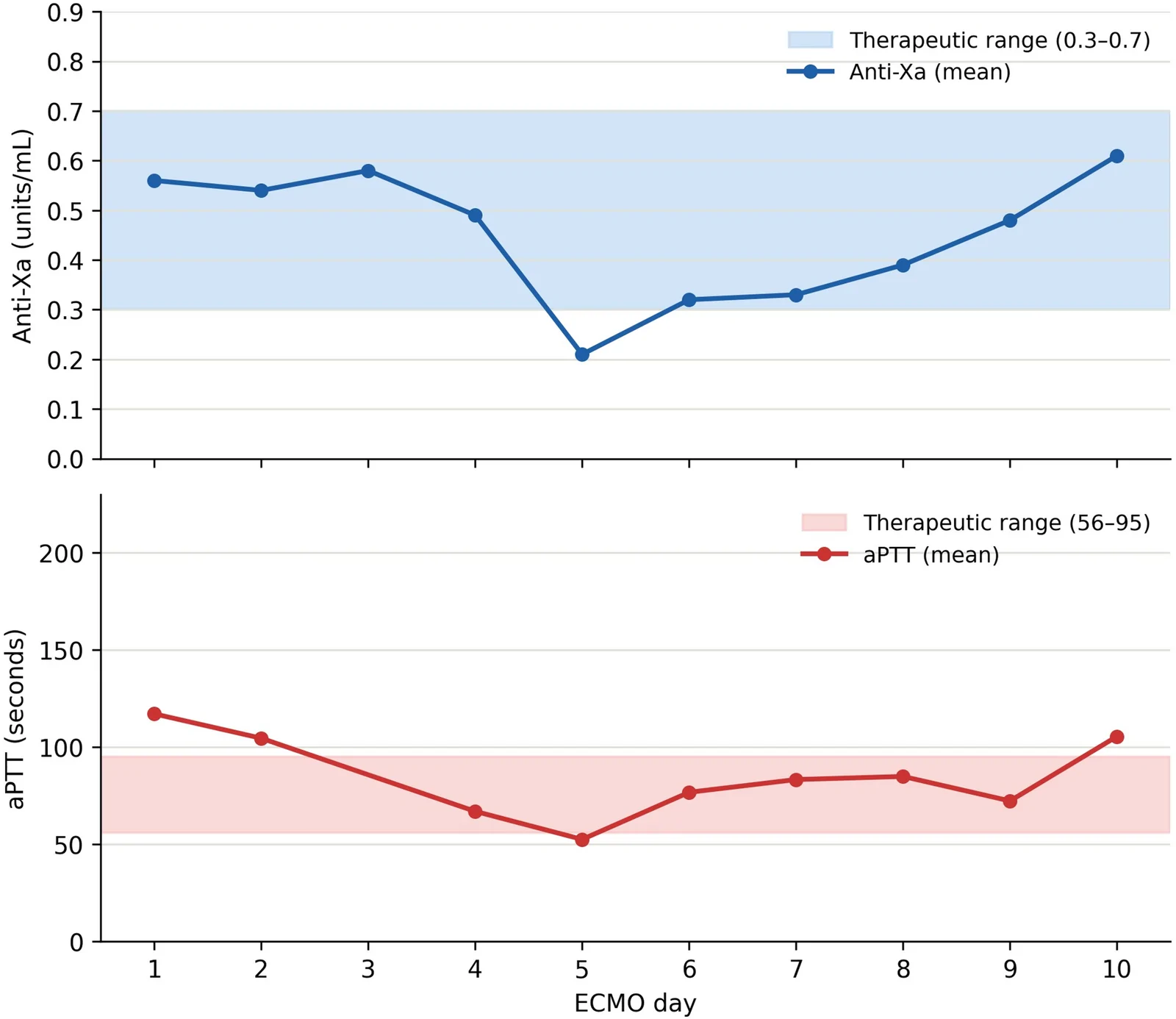
Hemoglobin and platelet trend for the duration of hospitalization

**Fig. 2:**
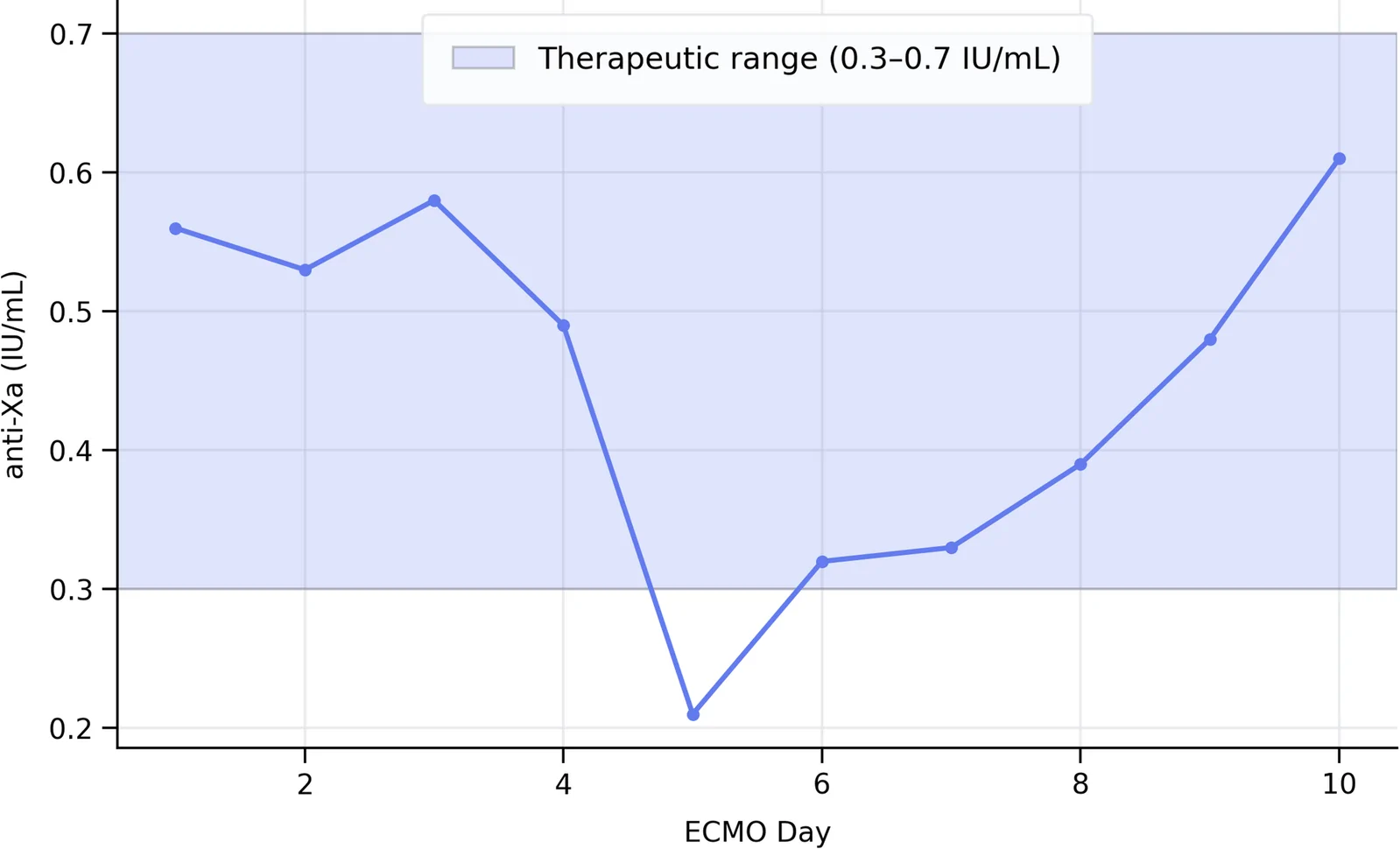
Trend of anti-Xa and aPTT levels for the duration of V-A ECMO support. Mean values utilized for multiple anti-Xa and aPTT levels obtained in a single day

The patient underwent a transfemoral TAVR-in-TAVR three days following ECMO cannulation. All accesses were obtained under ultrasound and fluoroscopic guidance using micropuncture needles on single pass, ensuring hemostasis to minimize blood loss. V-A ECMO was successfully weaned and decannulated 10 days after cannulation. The blood from the circuit was returned during decannulation and both cannulation sites were repaired primarily. His hemoglobin was 11.7 g/dL on the day of cannulation and 7.5 g/dL on the day of decannulation with a 6.3 g/dL nadir during his admission. Platelets were 180,000/mm^3^ on ECMO initiation and 92,000/mm^3^ when decannulated with a low of 73,000/mm^3^ while on the circuit (Fig. 1). The patient was therapeutically anticoagulated for the duration of support with V-A ECMO (Fig. 2).

At the time of discharge, TTE showed an LVEF 15–20%, Vmax 1.18 m/s, MG 12.7 mmHg. Repeat at 1 year showed an LVEF 30–35%, Vmax 1.95 m/s, MG 9 mmHg with trace aortic insufficiency. The only ECMO-related complication was polymicrobial infection of the cutdown site (*E. coli*, *S. marcescens*, *E. faecium*) requiring antibiotic therapy, re-exploration, and vacuum-assisted closure device placement. He did not receive any transfusions throughout his hospital stay despite multiple cardiovascular interventions. The patient was successfully discharged to a long-term acute care facility after 46 days of inpatient stay. On discharge, he required maximum assistance with activities of daily living with ongoing occupational and physical therapy. Neurologically the patient was oriented to person, place, time, and situation with no neurological deficit.

## Discussion

ECPR is a lifesaving intervention that is offered to patients who experience cardiac arrest and meet preset criteria. Despite the importance of this intervention, there are significant associated complications of which bleeding is paramount. The incidence of bleeding requiring blood transfusion during ECPR is conservatively estimated to be 9–40%, based on the definition of bleeding used [3]. This incidence reflects as high as 64% of ECPR patients requiring blood transfusions, needing up to 10 units packed red blood cells in addition to fresh frozen plasma and platelets as demonstrated in the SAVE-J II trial [4]. Associated risk factors include thrombocytopenia at presentation, older age, elevated D-dimer, surgical/central cannulation, and renal replacement therapy [4–6].

JWs can present clinical challenges, particularly in life-threatening scenarios that require invasive intervention. Though they or their decision makers may agree to intervention, blood product transfusion is prohibited based on their religious belief [7, 8]. An algorithmic approach to management is therefore imperative (Table 1) and therapeutic decisions should be made in conjunction with the patient and family, considering all relevant clinical details.

**Table 1:** Blood conservation management for V-A ECMO patients

INSERTION	Single puncture with ultrasound guidance
	Use of micropuncture needle/kit
	Miniaturize the ECMO circuit to minimize hemodilution
During support	Physical examination to check for bleeding
	Blood sampling with pediatric tube
	Minimize blood sampling
	Maintain lower end of aPTT to minimize amount of heparin
	Administration of IV erythropoietin, iron, cobalamin (VB12)
	Hematology consultation
Decannulation	Meticulous tissue handling
	Return circuit blood to the patient
	Use of topical hemostatic agent
	Cryoprecipitate, PCC, and single clotting factors if patient agrees to accept
Post decannulation	Physical examination to check for bleeding
	Minimize blood sampling
	Administration of erythropoietin
	Administration of IV iron
	Administration of cobalamin (VB12) supplements

aPTT, activated partial thromboplastin time; IV, intravenous; PCC, prothrombin complex concentrate; V-A ECMO; veno-arterial extracorporeal membrane oxygenation; VB12; vitamin B12

In emergency settings, optimization initiatives can be undertaken once the patient has been stabilized. Hematopoietic agents and nutritional optimization can be employed to stimulate production of native cell lines and prevent deficiencies that contribute to coagulopathy [2]. The key factor to successful outcomes is to ensure minimizing blood loss during interventions and reducing the rate of complications. Obtaining access during ECPR is challenging and is a critical moment to avoid complications and blood loss. Use of ultrasound-guided vascular puncture allows for reliable access and avoids multiple sticks that can lead to hematoma formation and limb ischemia. A two-person cannulation team allows for maintaining manual pressure at the access point during exchanges to minimize blood loss. When feasible, retrograde autologous priming of the circuit may be useful to avoid hemodilution [2].

Following cannulation, minimizing and consolidating blood draws with pediatric collection tubes is a conservative measure to avoid iatrogenic anemia. This can be a challenge as these patients are critically ill and on anticoagulation and thus require serial laboratory monitoring. Consideration can be given to lowering the therapeutic range set for monitoring anticoagulation effect. The current recommendation is an aPTT 1.5–2.5 times the pre-therapy baseline of the patient (60–90 s) or an anti-Xa level of 0.3–0.7 IU/mL [9]. However, to balance the risk of major bleeding with thrombotic complications, lowering the thresholds below what is recommended but achieving some degree of anticoagulation is recommended. This should largely be driven by institutional protocols and patient population factors.

Despite all the measures discussed, should a patient develop a hemorrhagic complication, rapid recognition and a predetermined multidisciplinary plan can be lifesaving. Systemic anticoagulation should be immediately discontinued, acceptable blood alternatives and hemostatic agents should be administered, and there should be a low threshold for intervention to achieve mechanical control of hemorrhage. Another consideration to limit bleeding complication is shortening the duration of V-A ECMO. Rapid liberation from V-A ECMO has been shown to be independently associated with bleeding complications [10]. As such, effort should be made to optimize patient’s hemodynamics to achieve rapid weaning. When the patient is ready to be weaned from ECMO, they should have unequivocal hemodynamics to avoid the need for re-intervention. In the setting of operative decannulation requiring cut downs and arterial repair, meticulous dissection with isolation and control of vessels should be performed for minimal blood loss. During the procedure, cell salvage should be used to re-infuse blood products if that is acceptable to the patient [7]. Lastly, the patient should be closely monitored post-operatively with frequent physical examination. Use of hematopoietic agents and minimizing blood tests should continue until the patient is recovered. With well-defined goals of care and an algorithmic approach, ECPR can be considered as a viable treatment option for JW patients. Given the limited number of reported cases of such utilization with successful outcome, this experience can be a reference for a traditionally challenging scenario.
